# Bilateral Vestibulopathy: Vestibular Function, Dynamic Visual Acuity and Functional Impact

**DOI:** 10.3389/fneur.2018.00555

**Published:** 2018-07-10

**Authors:** Ruben Hermann, Eugen C. Ionescu, Olivier Dumas, Stephane Tringali, Eric Truy, Caroline Tilikete

**Affiliations:** ^1^ENT and Cervico-Facial Surgery Department, Hôpital Edouard Herriot, Hospices Civils de Lyon, Lyon, France; ^2^INSERM U1028, CNRS UMR5292, Lyon Neuroscience Research Center, Equipe IMPACT, Lyon, France; ^3^University Lyon 1, Lyon, France; ^4^Department of Audiology and Otoneurological Evaluation, Hôpital Edouard Herriot, Hospices Civils de Lyon, Lyon, France; ^5^Société Française de Kinésithérapie Vestibulaire, Lyon, France; ^6^Department of Otology and Otoneurology, Hôpital Lyon Sud, Hospice Civils de Lyon, Lyon, France; ^7^Neuro-Ophthalmology Unit, Hopital Neurologique et Neurochirurgical P Wertheimer, Hospices Civils de Lyon, Lyon, France

**Keywords:** bilateral vestibulopathy, bilateral vestibular hypofunction, head impulse test, dynamic visual acuity, caloric tests, fall risk

## Abstract

**Introduction:** Patients suffering from bilateral vestibular hypofunction (BVH) often experience ataxia as well as visual instability. Even though progress has been made in vestibular testing, insights regarding vestibular deficit in BVH remain incomplete since no method allows evaluation of frequency ranges of vestibular sensors in a continuous way. The aim of our study was to give a detailed description of the level of vestibular deficit in different ranges of vestibular stimulation and an exhaustive evaluation of the functional impact including dynamic visual acuity (DVA) in a cohort of BVH patients in different etiologies.

**Methods:** We prospectively included 20 patients with chronic BVH. All patients underwent clinical evaluation and functional assessment including evaluation of their symptoms related to BVH, quality of life questionnaire and DVA in the horizontal and vertical plane. Patients underwent vestibulo-ocular reflex (VOR) testing using rotatory chair, caloric stimulation and video head impulse (vHIT) in the plane of the 6 canals, and cervical and ocular Vestibular evoked myogenic potentials.

**Results:** Mean rotatory VOR gain was 0.07 (*SD* = 0.07). Mean rotatory VOR gain during vHIT for the lateral, anterior and posterior canals was respectively < 0.28, < 0.34, and < 0.20. Mean loss of DVA in the 4 directions was >0.30 LogMAR. In our population fall frequency was significantly higher in patients with lower UniPedal Stance Test (UPST), higher Dizziness Handicap Inventory and Ataxia Numeric Scale (ANS) scores, as well as greater loss of upwards DVA. Patients with ototoxic BVH had a significantly higher residual VOR gain during vHIT in the anterior canal plane and lower DHI than other patients. In the general population anterior canal function was significantly higher than lateral or posterior canal function.

**Conclusions:** This study gives extensive descriptive results of residual vestibular function, DVA and quality of life in a population of patients suffering from severe BVH. UPST and ANS are good indicators for fall risk in case of BVH. Gentamicin induced BVH seems to have a lesser impact on quality of life than other etiologies.Anterior semi-circular canal function seems less deteriorated than lateral and posterior function.

## Introduction

Bilateral vestibular hypofunction (BVH) is a rare but disabling condition characterized by bilateral reduced or absent vestibular function, due to vestibular end organ and/or vestibular nerve dysfunction (1). Since February 2017, Barany society has established diagnostic criteria's (2) allowing a consensual definition of BVH. The estimated prevalence is of 28/100 000 in 2008 (3). BVH can be secondary to various etiologies and in over half of all cases no explanation is found (4). Predominant symptoms of BVH are imbalance (vestibular ataxia) and decreased dynamic visual acuity (DVA) and oscillopsia during head and body movements due to instability of gaze. Ataxia, especially in darkness or on uneven ground, is more constant than oscillopsia, the latter being reported in 40–81 % of patients suffering from BVH (4, 5). Nevertheless the impact on quality of life of both these symptoms can be very severe (6, 7). Patients suffering from BVH can have a very different perception of their handicap, with some mainly focused on their visual instability and other on their postural instability. The vestibular system combines five different neuro-epithelia capable of encoding head movements through mecanotransduction over a broad range of frequencies and over different types and directions of head movements. The 3 semicircular canals (SCC) respond to angular acceleration and the 2 otolithic organs respond to linear acceleration. Quantitative evaluation of vestibulo-ocular function, necessary to diagnose BVH, is usually done using caloric testing, rotatory chair testing, and video based quantitative head impulse testing (vHIT). Despite recent progress in vestibular testing, no tool allows clinicians to evaluate frequency range of vestibular sensors in a continuous way as it can be done in hearing loss. However, this may be approached in combining different methods of VOR evaluation with stimulus frequencies of 0.003, 0.25, and 5 Hz by using respectively caloric, rotatory chair and vHIT testing (8, 9). Even if a partial image of the vestibular system of our patients can be constructed, the establishment of a link between symptoms like ataxia and oscillopsia and a specific etiology, topography, or frequency of loss of function of the vestibular system remains to be answered.

This study proposed to perform in a prospective way both a multifrequential evaluation of vestibular function as well as an exhaustive evaluation of the functional impacts of BVH in a cohort of patients with different etiologies.

## Methods

This prospective study was held in the neuro-ophthalmology unit in University Hospital in Lyon between April 2016 and February 2017. All patients underwent neurological and vestibular assessment. The inclusion criteria were (1) age between 18 and 90. (2) Bilateral vestibular hypofunction shown on caloric irrigation (sum of bithermal [30°C/44°C] maximum peak slow phase velocity of 5°/s or less on each side), rotatory chair test (Sinusoidal stimulation in the horizontal plane, VOR gain lower than 0.2), and positive HIT (presence of refixation saccades). (3) Stability of BVH over at least 6 months. Patients with a corrected standard visual acuity lower than 0.3 LogMAR, other lesions leading to ataxia and/or oscillopsia, oculomotor palsy, or ocular instability in primary gaze position and patients with instability of the cervical spine and abnormal brainstem or cerebellum on MRI were not included. To rule out cerebellar and polyneuropathic ataxic disorders all patients underwent a detailed clinical neurological exam by a senior neurologist. Any abnormal findings led to further examination such as electromyoneurography.

A total of 20 patients were included in our study. The sex ratio was 1 with a median age of 60 years old (range: 22–80). Mean Duration of BVH was 9 years (range 1–32).

### Ethical issue

All patients were informed about the design and purpose of the study, and all gave their informed, written consent to the protocol. Approval was received from the National French ethical committee on human experimentation (n°160165B-32), in agreement with French law (March 4, 2002) and the Declaration of Helsinki. The study was registered in a public trials registry (ClinicalTrials.gov Identifier: NCT02753179).

### Assessment of vestibular ataxia and oscillopsia

Ataxia was quantified using UniPedal Stance Test (UPST) measured in seconds. The test was considered normal if unipedal stance could be maintained longer than 10 s. Subjects had multiple trials on both legs and only the best result was kept. Subjects were also asked how many times a year they had fallen after the onset of the BVH. To assess self-perceived severity of ataxia we asked subject to evaluate their postural instability on a simple Ataxia Numeric Scale (ANS) from 0 (no imbalance) to 10 (worst possible instability).

Oscillopsia was assessed with the Oscillopsia Severity Questionnaire (OSQ) developed by Guinand et al. (5) (Annex I). We also used a simple Oscillopsia Numeric Scale (ONS) from 0 (no oscillopsia) to 10 (worst possible oscillopsia).

Collected data also included: age, sex, date of onset, patient history, adjuvant treatment, MRI, vertigo, and underlying etiology.

### Quality of life

Quality of life was assessed using two different questionnaires which were filled out by the patient or with the help of an examiner during the study. We used the Short Form - 36 (SF-36) (10) to assess global quality of life and the Dizziness Handicap Inventory (DHI) (11) to assess the quality of life linked to vestibular dysfunction (Annex I).

### Functional visual evaluation

Standard visual acuity was first evaluated with best eye correction using Monoyer chart.

Static and dynamic visual acuities were then evaluated using a commercialized device (Framiral, Grasse, France). The method for this evaluation has been described in detail in a previous study (12).

**Figure 1:**
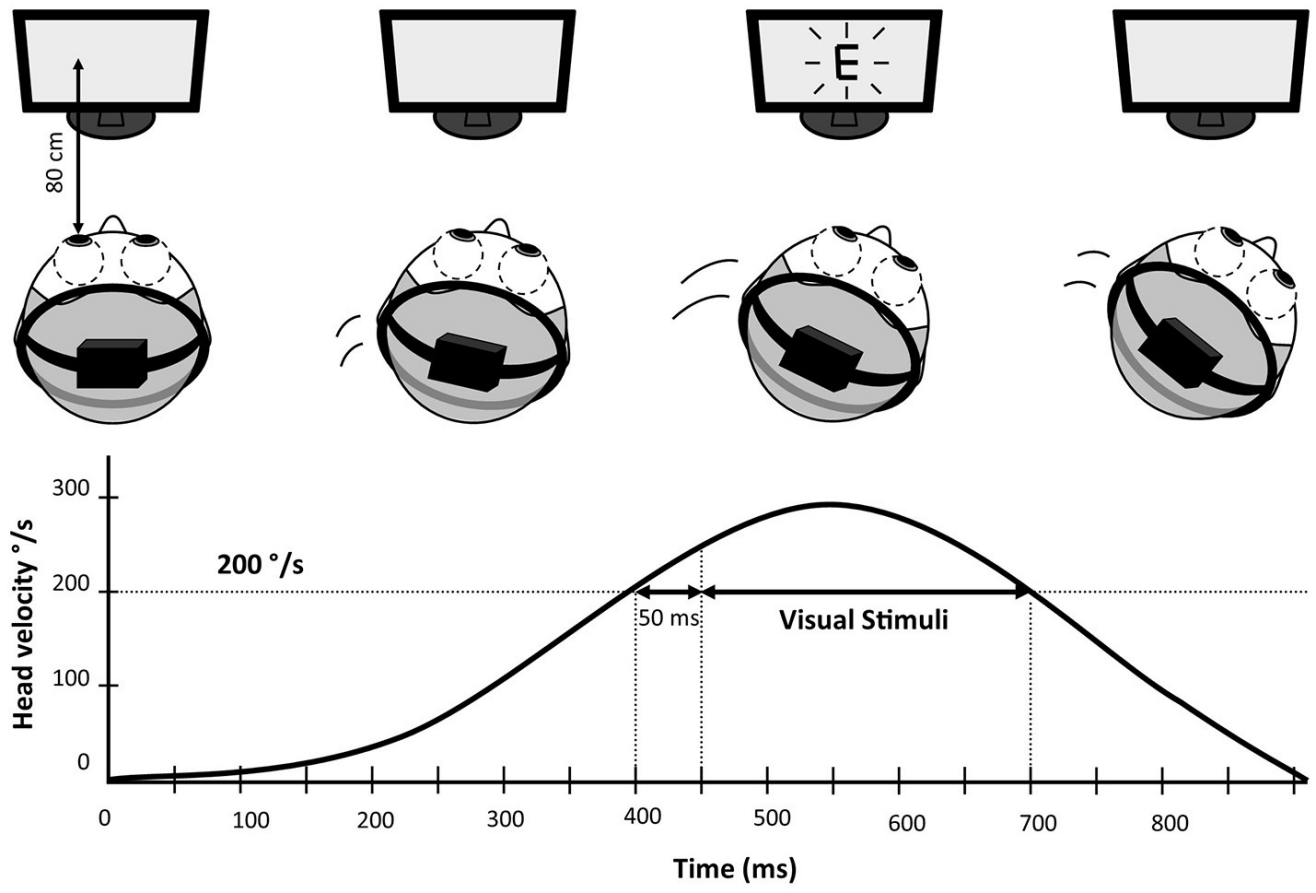
Schematic representation of Dynamic Visual Acuity (DVA) measurement. The subject wears a lightweight helmet equipped with a 9 axis motion tracking device recording head speed with a 100 Hz frequency. The visual stimuli (letter “E” in this example) is enslaved to head rotations and only appears if the speed of the head rotation was within 200–300°/s for more than 50 ms.

DVA was measured in lateral (left and right) (Figure 1) and vertical (up and down) directions of head rotations. This test was done with eyeglasses if needed. Both DVA and change from static to DVA (visual acuity loss) were analyzed.

### Video head impulse test (vHIT)

The vHIT was recorded using a lightweight portable vHIT device (Hardware: ICS Impulse, GN Otometrics, Taastrup, Denmark, Software: Otosuite® Vestibular software). The method for this evaluation as well data analysis has been described in detail in a previous study (12).

The vHIT was performed 15 mn after DVA testing. Outward horizontal and vertical head impulses were performed by experienced examiner standing behind the patient. For the evaluation of the horizontal SCCs the head was tilted forward to align the plane of the horizontal SCCs with the horizontal plane. A minimum of 5 valid horizontal head impulses were realized in each direction with a target speed > 200°/s. For the evaluation of the vertical SCCs vestibulo-ocular function, the head was turned 40° to the side to align the horizontal gaze direction with the plane of the simulated SCC pair [right anterior-left posterior (RALP) or left anterior-right posterior (LARP)] (13). A minimum of 5 valid vertical head impulses were realized in each direction with a target speed > 150°/s. Head and eye velocity data were then exported in CSS format for off line analysis.

### Cervical and ocular vestibular evoked myogenic potentials (VEMP)

Cervical (cVEMPs) and ocular VEMPs (oVEMPs) were used to assess otolithic function (ICS chartr EP 200, GN Otometrics, Taastrup, Denmark). Detailed methodology for this evaluation has been described in Annex II.

### Rotational chair testing

#### Eye movement recording

Subjects were seated on a servo-controlled rotating chair with the head in erect position. The subjects were in complete darkness. A goggle with an infrared light and video camera was mounted in front of the subject's right eye to record horizontal eye movements. Calibrations were made by having subjects sequentially fixate a series of visual targets (1°) positioned 15° apart at a distance of 1.8 m. Eye images were digitized (50 Hz) and eye velocity was computed in a head frame of reference using a commercial off-the-shelf software (VNG Ulmer, Synapsys SA, Marseille, France).

#### Stimulations

Sinusoidal rotatory chair testing was done (burst of sinewave for 30 s) at 0.25 Hz with 60°/sec peak chair velocity to assess:

- Visually enhanced vestibulo-ocular reflex (VVOR) by recording eye movements with viewing
- Vestibulo ocular reflex (VOR) by recording eye movements without viewing
- Cervico-ocular reflex (COR) by recording eye movements in the dark and maintaining the head of the patient fixed in space as to create a rotation of the head on the trunk in vertical axis.

#### Eve movement analysis

The velocity of eye slow phases and chair movements were analyzed using Fourier analysis and at stimulation frequency (0.25 Hz). VVOR, VOR, and COR gains were measured by dividing slow phase eye velocity by chair velocity.

### Caloric testing

#### Eye movement recording

Eye movements were recorded with the method described in the Rotational chair testing paragraph. For caloric testing, subjects were in a supine position with their head inclined forward 30°.

#### Stimulations

Bithermal caloric testing was done using alternatively 60 ml of cold (30°C) and hot (44°C) water irrigation during 30 s in the right, then the auditory canal. Nystagmus was recorded during 90 s after the end of irrigation using video-oculography (VNG Ulmer, Synapsys SA, Marseille, France).

#### Eve movement analysis

Absolute value of peak slow phase velocity of nystagmus was measured for cold and hot water irrigation and summed on each side.

### Statistical analysis

All data were stored and analyzed using Statistica 10 (Softonic®, Barcelona, Spain). Statistical analysis was done using Spearman's rank order correlation as well as linear regression and Mann Whitney's U-test. All tests were two-tailed and *P*-values < 0.05 were considered significant.

## Results

### Etiologies

**Figure 2:**
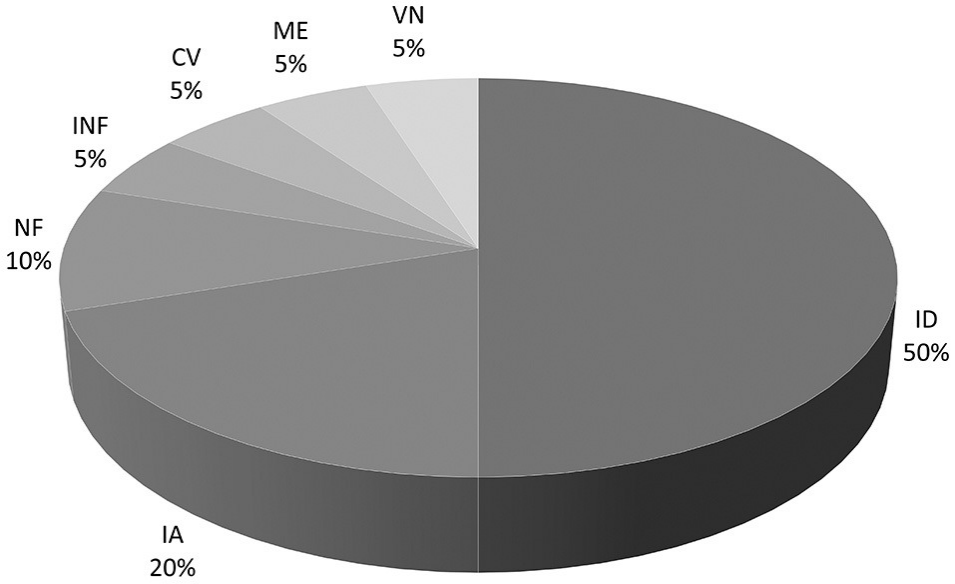
Pie chart of etiologies of BVH. ID, Idiopathic; IA, Iatrogenic; NF, Type 2 Neurofibromatosis; INF, Bilateral middle ear infection; CV, Cochleo-Vestibular hypofunction: progressive BVH associated with progressive profound bilateral hearing loss; ME, Meniere's disease; VN, Bilateral vestibular neuritis.

Ototoxicity was the most common identified cause of BVH with 4 patients (20%). All 4 cases were related to systemic gentamicin treatment. In half of all cases no cause could be found. Etiologies are shown in Figure 2.

### Hearing

Three patients had a concomitant moderate to severe neurosensorial hearing loss. One had a mild conductive hearing loss. The other 16 patients had normal hearing in accordance with their age.

### Vestibular function

Results of rotatory chair and caloric testing as well as otolithic function are shown in Table 1. For rotatory chair mean VVOR gain was 0.61 (*SD* = 0.19), mean VOR gain was 0.07 (*SD* = 0.07), and mean COR gain was 0.37 (*SD* = 0.20). Mean sum of bithermal maximum peak slow phase velocity was 0.90°/s (*SD* = 1.19) on the right side and 1.07°/s (*SD* = 1.19) on the left side. There was no significant difference between left and right side. There were no differences with regards to age in VVOR, VOR, or COR gains and caloric response. There was a tendency for a positive correlation between longer duration of BVH and higher COR (*p* = 0.056) but no correlation was found between patient age and COR (*p* = 0.73).

**Table 1:** Results of caloric, rotatory chair and otolithic testing.

**Patient**	**Age (range)**	**Duration (years)**	**Caloric right (^°^/s)**	**Caloric left (^°^/s)**	**Chair VVOR**	**Chair VOR**	**Chair COR**	**cVEMP**	**oVEMP**
1	76–80	1	2.70	4.90	0.93	0.13	0.03	R–L–	R–L–
2	60–65	7	0.20	2.60	0.88	0.18	0.27	R+L+	R–L–
3	60–65	32	0.50	0.50	0.80	0.18	0.71	R–L–	R–L–
4	66–70	2	0.10	0.50	0.92	0.17	0.70	R–L+	R–L–
5	56–60	2	1.90	1.40	0.51	0.04	0.25	R–L+	R–L–
6	66–70	10	3.50	2.00	0.65	0.19	0.65	R+L+	R–L–
7	50–55	4	0.50	1.60	0.71	0.09	0.65	R+L+	R–L–
8	70–75	10	0.60	0.70	0.72	0.01	0.51	NA	NA
9	40–45	31	0.20	0.80	0.96	0.06	0.52	R–L–	R–L–
10	76–80	5	1.40	0.20	0.54	0.03	0.51	NA	NA
11	80–85	9	0.50	0.50	0.36	0.03	0.43	R–L–	R–L–
12	46–50	11	1.80	0.80	0.99	0.01	0.25	R+L+	R–L–
13	50–55	2	0.20	0.10	0.37	0.04	0.10	R–L–	R–L–
14	46–50	4	1.50	2.80	0.64	0.16	0.52	R–L–	R–L–
15	60–65	16	0.80	0.40	0.62	0.06	0.40	R–L–	R–L–
16	30–35	4	1.40	1.10	0.82	0.20	0.25	R+L–	R+L+
17	70–75	12	0.00	0.30	0.64	0.02	0.36	R–L–	R–L–
18	70–75	15	0.20	0.00	0.65	0.00	0.30	R+L+	R+L+
19	20–25	3	0.00	0.20	0.78	0.04	0.29	R–L–	R–L–
20	66–70	3	0.00	0.00	0.54	0.02	0.02	R+L+	R+L–
Mean	60.00	9.15	0.90	1.07	0.70	0.08	0.39		
*SD*	16.2	1.99	0.96	1.19	0.18	0.07	0.20		

*Caloric, sum of bithermal max peak slow phase caloric induced nystagmus; Chair, rotatory chair testing; VVOR, visually enhanced vestibulo-ocular reflex; VOR, Vestibulo-Ocular Reflex; COR, Cervico-Ocular Reflex; cVEMP and oVEMP, cervical and ocular vestibular evoked myogenic potentials; R, Right; L, Left; +, identifiable response during VEMPs; −, no identifiable response; NA, no possible assessment of otolithic function*.

**Figure 3:**
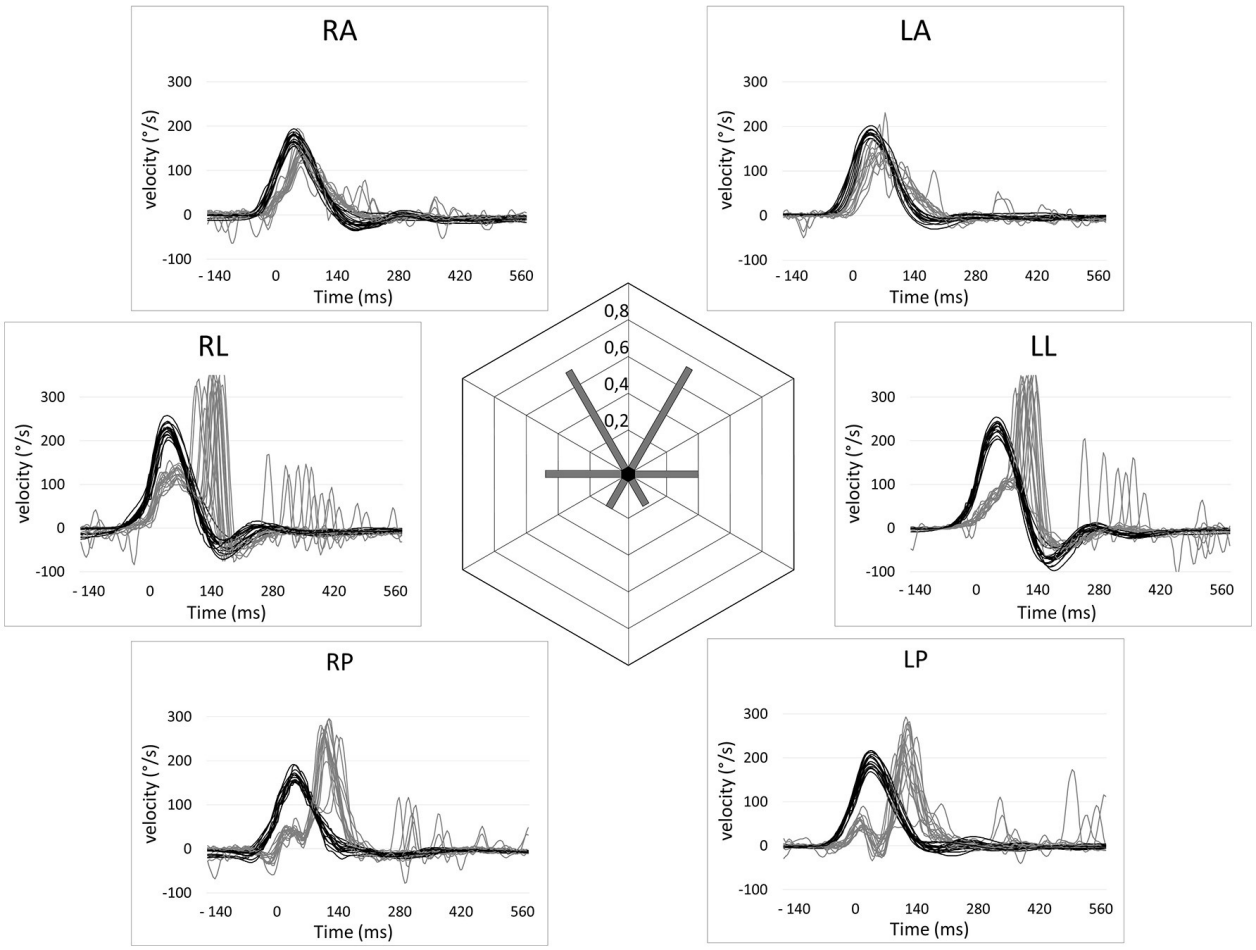
Video head impulse test recordings of a patient with a gentamycin-induced bilateral vestibular hypofunction showcasing a relative sparing of the anterior semi-circular canal function. Eye velocities (gray lines) and head velocities (black lines) relative to time are shown for the six semi-circular (RA, Right anterior; LA, Left anterior; LL, Left lateral; LP, Left posterior; RP, right posterior; RL, right lateral). Residual VOR gains are shown in the center.

**Figure 4:**
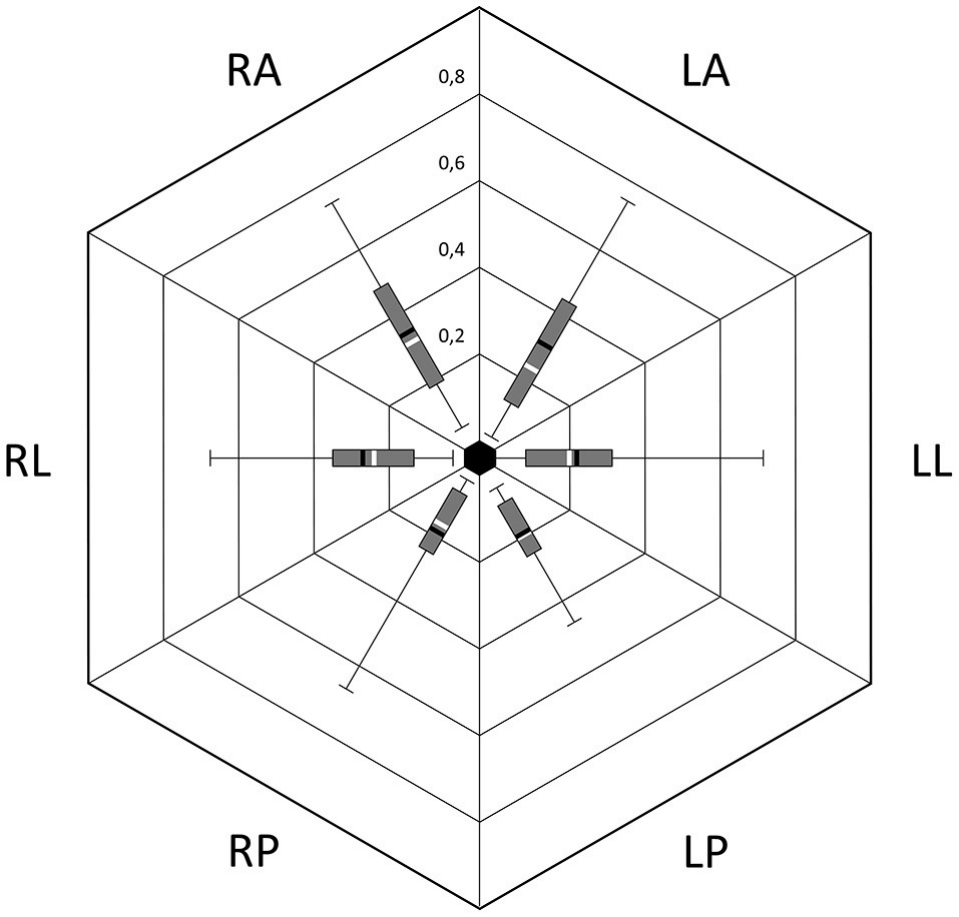
Distribution of VOR gains during vHIT; Box, 1st and 3rd quartile; T-Bars, edge values; Black bar, mean; White bar, median; RA, Right anterior; LA, Left anterior; LL, Left lateral; LP, Left posterior; RP, Right posterior; RL, Right lateral.

**Figure 5:**
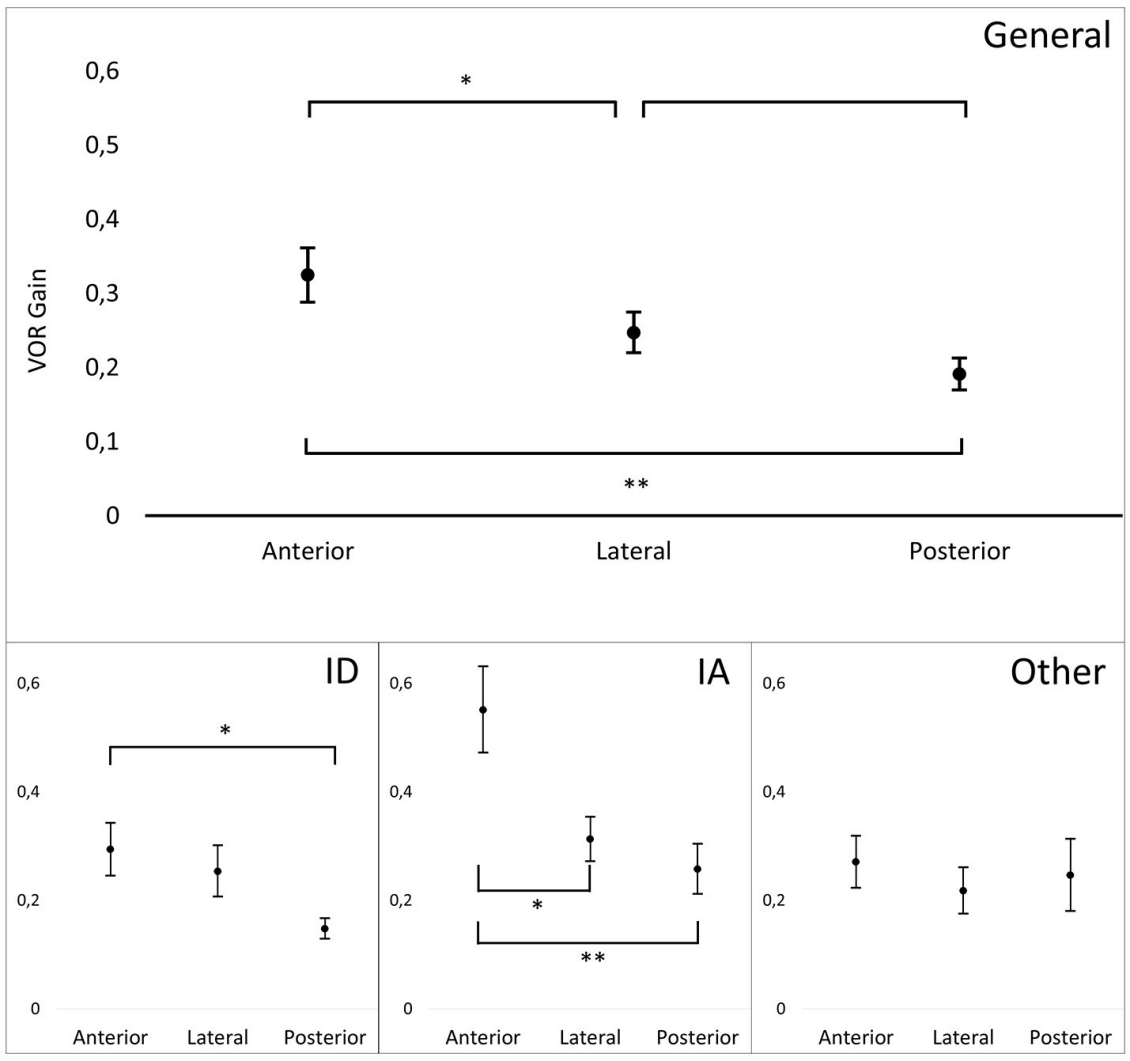
Mean VOR gains for pairs of canals during vHIT in different patient groups. General, General population (*n* = 20); ID, patients with Idiopathic BVH (*n* = 10); IA, Patients with gentamicin induced BVH (*n* = 4); Other, Other etiologies (*n* = 6). T bar represents standard error. Significant differences are indicate by one (*p* < 0.05) or two (*p* < 0.01) asterisks.

Mean VOR gains during vHIT were the following: left lateral: 0.22 (*SD* = 0.17); right lateral: 0.28 (*SD* = 0.18); left anterior: 0.31 (*SD* = 0.26); right anterior: 0.34 (*SD* = 0.21), left posterior: 0.19 (*SD* = 0.11); right posterior: 0.19 (*SD* = 0.16). There was no significant difference of VOR gain between left and right side for lateral, posterior and anterior SCCs. In the overall group, cumulative residual VOR gain in the anterior SCCs was significantly higher compared to lateral SCCs (0.32 vs. 0.25, *p* < 0.05) and posterior SCCs (0.32 vs. 0.19, *p* < 0.01). This was also true for the subset of patients suffering from gentamicin induced BVH (*p* < 0.05). For patients suffering from idiopathic BVH the only significant difference was between anterior and posterior SCC gain (0.29 vs. 0.15, *p* < 0.05). The difference between lateral ant posterior SCC gains was not significant in the overall group and in the sub-groups of patients. Distributions of the different VOR gains in one patient are shown in Figure 3 and for the overall patient group in Figure 4. Mean VOR gains of all 3 SCC pairs in the overall group and in some sub-groups are shown in Figure 5. VOR gain during rotatory chair testing was not correlated with residual VOR gain for lateral canals during vHIT.

### VEMP

One patient had bilateral intact cVEMP and oVEMP and nine had bilaterally absent VEMPs. cVEMPs were present in both ear for 6 patients and in one ear for 3 patients. oVEMPs were present in both ear for 2 patients and in one ear for 1 patients. For two patients, assessment of the otolithic function was no possible.

### Visual acuity

**Figure 6:**
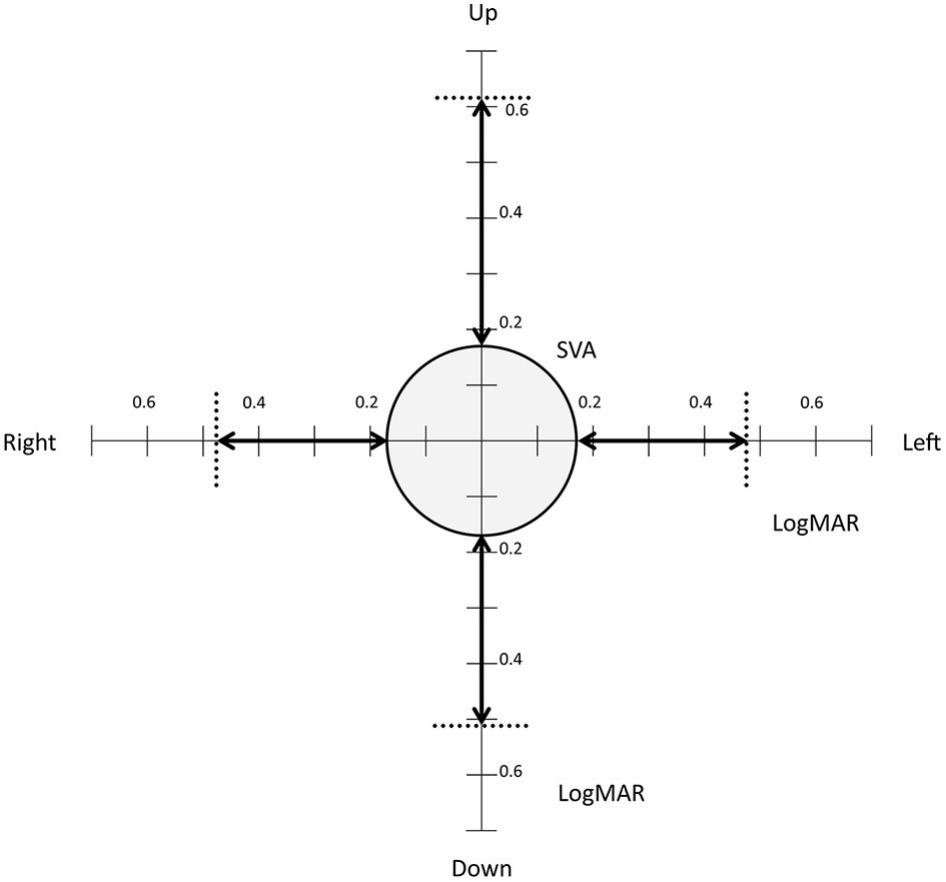
Static and dynamic visual acuity. The circle shows mean static flashed visual acuity (0.17 LogMAR). The dotted lines show mean dynamic visual acuity during head turns to the right, left, up and down. The arrows show loss of Visual Acuity due to head movement in the 4 directions.

Mean static visual acuity with flashed optotypes was 0.17 LogMAR (0 LogMar is equivalent to a 20/20 vision). Two patients did not manage to undergo complete visual acuity testing due to difficulties in reaching head rotation speeds between 200 and 300°/s in the vertical plane. Mean DVA in the 4 directions was 0.48 LogMAR (*SD* = 0.18), 0.47 LogMAR (*SD* = 0.18), 0.61 LogMAR (*SD* = 0.23), and 0.51 LogMAR (*SD* = 0.21) for respectively left right up and down. Dynamic visual acuity was significantly decreased as compared to static visual acuity in all 4 directions (*p* < 0.001). Loss of Visual Acuity due to head movement was 0.30 (*SD* = 0.13), 0.30 (*SD* = 0.12), 0.45 (*SD* = 0.19), and 0.35 (*SD* = 0.15) for respectively left right up and down. There was no significant difference in DVA between directions. Detailed results of DVA and DVA loss are shown in Figure 6. Age was negatively correlated with DVA in all four directions (*p* < 0.05).

### Functional impact and quality of life

All patients reported symptoms related to vestibular ataxia. Falls associated with balance problems were present in 11 patients, with 5 of these patients falling more than twice a year. Mean unipedal stance test was 4.9 s (*SD* = 3.45). Mean Ataxia Numeric Scale (ANS) was 6.1/10 (*SD* = 1.52). UniPedal Stance Test (UPST) was significantly lower in the group of patients who had experienced falls (3.3 s) when compared to those who hadn't (6.9 s) (*p* < 0.05). The Dizziness Handicap Inventory (DHI) and the Numeric Scale for Ataxia (ANS) were higher in the fall group (DHI: 54.3; ANS: 6.9) than in the other group (DHI: 39.1; ANS: 5.1) (respectively *p* < 0.05 and *p* < 0.01). Finally upward DVA was significantly worse (*p* = 0.01) and loss of upwards DVA significantly higher for the fall group (*p* < 0.05) when compared to the other.

Mean Oscillopsia numeric scale (ONS) rating was 6.25/10 (*SD* = 2.29) and mean Oscillopsia Severity Questionnaire (OSQ) scores were 3.0/5 (*SD* = 0.92). Two out of the 20 patients had no oscillopsia with regards to ONS (0 and 1). All other patients had an ONS over 5. When using OSQ, 3 patients had a score lower than 2. There was a correlation between scores of Oscillopsia Severy Questionnaire (OSQ) and Numeric Scale for Oscillopsia (*R*2 = 0.276, *p* < 0.05) but only OSQ was positively correlated with mean DHI score (*R*2 = 0.295, *p* < 0.05). There was no statistical correlation between oscillopsia and DVA or loss of VA in our population.

Mean DHI Scores were 47.5 (*SD* = 16.8). Twelve patients had a score between 30 and 60 and five patients had a score >60. Mean Sub-scores were 12.7, 18.4, and 16.4 for respectively physical, emotional, and functional sub-score. Mean MOS-SF 36 score were 44.3 (*SD* = 9) and 40.6 (*SD* = 10.5) for respectively physical and mental component. Patients with ototoxic BVH had lower DHI scores (31) when compared to idiopathic (51) or when compared to other causes of BVH (53) (both *p* < 0.05).

## Discussion

The goal of this study was to give a detailed description of the residual vestibular functions and DVA of patients with bilateral vestibular hypofunction as well as to demonstrate possible correlations between loss of specific vestibular functions, functional impact of BVH, and other epidemiologic factors. The epidemiology of our population, the objective vestibular functions and finally the functional consequences of BVH relative to etiologies will be discussed.

As this study started in April 2016, our inclusion criteria for BVH are not identical to those suggested by the Barany Society published in 2017 (2). Our criteria appear more constraint but importantly all patients in this study fit the definition of BVH according to these new criteria. Our population is representative of BVH in terms of age and gender when compared to other studies (4, 14, 15, 16). For half of our patients no etiology could be identified and the most frequent identified cause was ototoxic aminoglycosides (20%). Both of these results are in accordance with results found in the literature as 30–50% of all cases of BVH seem to be idiopathic (1, 4) and 13–19% due to ototoxic drugs and especially aminoglycosides (4, 16). Only one of our patients was diagnosed with Meniere disease which is less than what had been reported prior to this work (7–16%) (1, 4, 16). This can be due to more selective inclusion criteria used in our study as our patients had a confirmed deficit in caloric, rotatory chair, and video head impulse testing. Indeed it seems that prevalence of Meniere's disease in BVH is higher when diagnostic criteria's for BVH are less restrictive (1, 4, 16). In our population 80% of patients had a normal hearing in accordance with their age. This is higher than what has previously been published (34–49%) (1, 7, 15) and could be partly due to the low prevalence of Meniere disease in our study. We also excluded patients with other lesions leading to ataxia and/or oscillopsia such as cerebellar disorders. These exclusion criteria allows us to attribute ataxia and decreased quality of life in our patient mostly to BVH.

Only four of our patients had a mean peak slow phase velocity greater than 2°/s during caloric irrigation. VOR gain during rotatory chair testing was not correlated with residual VOR gain for lateral canals during vHIT. This could be due to frequency specific VOR deficit. Another explanation could be vestibular habituation of VOR for medium frequencies (rotatory chair testing) after repeated rotational stimulation. This habituation can lead to a long lasting decrease of VOR gain for these frequencies (17).

During vHIT, VOR gains were not automatically calculated using the supplied software but manually calculated. The reason for this choice was that, especially for low VOR gains, vHIT are susceptible to artifacts due to goggle slippage and very early saccades. This was obvious in one patient who had undergone a vestibular neurectomy due to vestibular schwannoma and for whom the VOR gains for lateral, anterior, and posterior canal on the operated side were respectively 0.41, 0.56, and 0.76 with the supplied software and 0.25, 0.05, and 0.03 with manual calculation. Even though manual correction was not able to completely remove artifacts, results were still closer to the expected complete absent of VOR gain in this case. Artefacts can also be avoided by using tools such as suppression head impulse test (SHIMP) (13) but we did not have access to SHIMP testing for this study. Mean vHIT VOR gain in our population was lower than in previous studies for anterior, lateral, and posterior SCC function with residual VOR Gain being respectively 0.32 vs. 0.73, 0.25, vs. 0.54 and 0.19 vs. 0.5 (17). This is probably also due to more restrictive patient selection on our part as their inclusion criteria were limited to pathological vHIT. In our population anterior SCC function was significantly less deteriorated than lateral and posterior function with regards to residual VOR gain during vHIT. This was true in our general population as well as in patients suffering from gentamicin induced BVH. A previous study has already highlighted disease-specific sparing of the anterior SCCs in BVH (18). These authors suggested that this could be due to gravity induced accumulation of gentamicin in the posterior and lateral SCCs as these are located below the anterior SCC when the patient is lying down. Only one patient had bilateral intact cVEMP and oVEMP and nine had bilaterally absent VEMPs. Saccular and utricular function is often altered in BVH (19, 20) though the consequences of loss of otolithic function in BHV is still unclear. Nevertheless VEMP could be used to monitor disease progression (2).

Falls and fall related injuries are a recurrent challenge for patients suffering from BVH due to increased fall risk not only when compared to the general population (31-fold) (3) but also when compared to patients with peripheral vertigo and dizziness (21). In our population 55% of patients had experienced falls after onset of BVH. Not age but decreased UPST and higher DHI and ANS were associated with a history of falls. UPST and ANS seem especially relevant as they are easy to perform and not time consuming. We propose a UPST ≤ 4 s and a ANS score ≥ 6 to be good indicators of high fall risk as both of these indicators have a sensitivity of 82% and a specificity of 78% in our population. Upwards DVA and Loss of DVA were worse in patients with a history of falls. Whether decreased vision during upward head movement participates in falls remains to be questioned. Other risk factors for fall that have been identified in BHV include increase in temporal gait variability, especially at slow walking speeds, and a concomitant peripheral neuropathy (22).

Out of the 18 patients who managed to undergo complete testing of the Dynamic visual Acuity (DVA) 72% had a decrease of ≥ 0.2 LogMAR in DVA in all four direction which is considered pathological (2). All patients had a decrease of ≥ 0.2 LogMAR in at least one direction. We did not find any correlation between decreased DVA and quality of life. Furthermore there was no correlation between severity of oscillopsia (with regards to OSQ and NSO) and DVA which is in accordance with a previously published study (5). Even though both symptoms are a consequence of deficient VOR and slippage of the image on the retina, different adaptation mechanisms may develop in these two different symptoms. Development of tolerance to the slippage of images on the retina may improve perception of oscillopsia. This tolerance to retinal slip has been demonstrated by showing a decreased ability to detect coherent motion in BVH patients (23). This perceptive habituation is supposed to involve the middle temporal visual motion processing area. Another study highlighted the great variability of oscillopsia in patients with BVH, and even showed that patients with the greatest retinal slip were the least handicapped by oscillopsia, suggesting the development of tolerance to the movement of images on the retina (24). On the other hand DVA in BVH might be improved by transient stabilization of gaze during head thrust due to compensatory saccades of short latency called covert-saccades (CS) (25). These saccades are thought to have a positive impact on DHI (26, 27). In a recent study we have brought to light that not only presence of CS but their latency and amplitude have an impact on DVA (i.e., the shorter the latency and the bigger the CS, the better the DVA) (12). DVA, but not oscillopsia, has also been shown to improve after specific vestibular rehabilitation involving high speed head rotations (28, 29). These differences suggest that specific treatment options should be suggested to patients depending on their visual complaint.

In our population 85% of patients complained of oscillopsia. Previous studies have reported oscillopsia in 40–81% of cases (4, 5). OSQ but not NSO was correlated with DHI in our study suggesting the former to be more relevant to quantify Oscillopsia. The link between DHI and OSQ had already been highlighted (5). In the Oscillopsia severity questionnaire, we did not find the question relative to oscillopsia during bicycle riding suitable since most of our patients (65 %) were not able to ride a bike anymore.

BVH has a negative impact on quality of life as documented by lower SF-36 scores and higher DHI score (7). According to the DHI, 60% of our patients had a moderate and 25% a severe self-perceived handicap. We found a lower self-perceived handicap in patients with gentamicin induced BVH. To our knowledge these had not been shown yet. Studies have highlighted that in animal models, intratympanic gentamycin injection leads to a selective loss of Type I hair cells (30). Furthermore it has been documented that maculae are less sensitive to gentamicin, as shown by a lesser neuroepithelial thinning when compared to cristae, leading to a relative sparing of the otolithic organs (31). Type I hair cells with their irregular discharge pattern and greater sensitivity during large head accelerations are thought to be more relevant for initiation of VOR through rapid detection of head movement (32, 33). Furthermore VOR gain has been shown to be better correlated to presence of type I than type II hair cells (34). On the other hand Type II hair cells seem to provide a signal over a broader range of frequencies and help maintain VOR during sustained head rotation (9). This relative sparing of type II cells and otolithic organs could explain why, in our population, patients with gentamicin induced BVH have a lesser perceived handicap than other patients. However we did not find any differences between etiologies in saccular or utricular function, as reflected cVEMP and oVEMP testing. Further studies with a bigger cohort of patients are needed to test these hypothesis.

In our population, DVA in all four directions worsened with age in our population. The negative impact of age on DVA had already been shown in healthy subjects (35). In case of BVH it is unclear whether age related worsening of DVA could be due, besides decreased visual capacities, to decreased neural plasticity i.e. capacity to compensate deficient VOR by covert-saccades.

## Author contributions

RH and CT contributed to planning of the study, acquisition of data, statistical analyses, interpretation, and writing of the manuscript. EI contributed to planning of the study, reviewed the manuscript, and edited the manuscript. OD contributed to the planning of the study and acquisition of data. ET and ST reviewed and edited the manuscript.

### Conflict of interest statement

The authors declare that the research was conducted in the absence of any commercial or financial relationships that could be construed as a potential conflict of interest.
