# Supplementary material for: Bilateral Vestibulopathy: Vestibular Function, Dynamic Visual Acuity and Functional Impact
Source: Front Neurol. 2018 Jul 10;9:555. doi: 10.3389/fneur.2018.00555 (PMC6048872; doi:10.3389/fneur.2018.00555)
Supplement: Supplementary file 1 [file Table_1.DOCX]

Annex I : Questionnaires

Oscillopsia Severity Questionnaire (OSQ).

This questionnaire is composed of 9 items scored 1 (never), 2 (seldom), 3 (sometimes), 4 (often) or 5 (always). Each score is averaged, giving an oscillopsia severity score ranging from 1 (no oscillopsia) to 5 (worst possible oscillopsia).

Short Form – 36.

This questionnaire is composed of 36 items measuring 8 multi-item variables:

- Physical functioning (PF)
- Role physical (RP)
- Bodily pain (BP)
- General health (GH)
- Vitality (VT)
- Social functioning (SF)
- Role emotional (RE)
- Mental health (MH).

For each variable item scores are coded, summed, and transformed on to a scale from 0 (worst possible health state measured by the questionnaire) to 100 (best possible health state). We used the validated French version of the SF-36 [11,12].

Dizziness Handicap Inventory (DHI).

This questionnaire is composed of 25 items evaluating impact of dizziness and unsteadiness on quality of life. It is grouped onto 3 subscales:

- Emotional
- Functional
- Physical

Each item can be answered by “No” (0 points), “Sometimes” (2 points), and “Yes” (4 points). The end result of the global score can range from 0 (absence of perceived handicap) to 100 (worst perceived handicap). We used the French version of the DHI [14].
