# Supplementary material for: Bilateral Vestibulopathy: Vestibular Function, Dynamic Visual Acuity and Functional Impact
Source: Front Neurol. 2018 Jul 10;9:555. doi: 10.3389/fneur.2018.00555 (PMC6048872; doi:10.3389/fneur.2018.00555)
Supplement: Supplementary file 2 [file Table_2.DOCX]

Annex II : Cervical and ocular Vestibular evoked myogenic potentials (VEMP).

The sound stimulations were 6 ms long 500 Hz tone burst at 94 dB nHL with a repetition frequency of 5.1 Hz. Sounds were transmitted by air conduction using a calibrated head set. For cVEMPs, recording of sternocleidomastoid (SCM) muscle evoked activity was performed using active electrodes placed on the upper half of the SCM with left and right electrodes being symmetrical in relation to the neck. The reference electrodes were placed on the sternoclavicular joint. On each side a third electrode was placed 2 cm below the active electrode following SCM trajectory to monitor myogenic activity in the SCM muscle during testing. The ground electrode was placed on the forehead. Subjects were in a seated position with the head placed to the opposite side of the tested ear and were asked to contract SCM by turning their head towards the tested ear against resistance (the hand of an examiner). They could control their SCM contraction by viewing a monitor indicating if the contraction was too low (blue light), just right (green light: between 60 and 80 µV), or too high (red light). Signals were only recorded when the muscle contraction was just right. The session ended when 150 signals per side were recorded and averaged. Each side was tested twice. cVEMPs were considered present when positive (P13) and negative (N23) peaks could be identified. If present, P13-N23 amplitude was corrected according to the mean EMG activity. For oVEMPs, recording of ocular muscle evoked response was performed using active electrode placed in line with the pupil in primary position just below the lower lid of the eye. The reference electrode was placed 2 cm below the active one. The ground electrode was placed on the forehead. Subjects were in a supine position and were asked to maintain a maximal upward gaze as long as sound stimulation was present. 150 signals per side were recorded and averaged. Each side was tested twice. oVEMPs were considered present when negative (N11) and positive (P15) peaks could be identified.
